# Protein scaffolds: A tool for multi-enzyme assembly

**DOI:** 10.1016/j.btre.2021.e00670

**Published:** 2021-09-06

**Authors:** Shubhada Gad, Sonal Ayakar

**Affiliations:** Department of Biotechnology, Institute of Chemical Technology - IndianOil Odisha Campus Bhubaneswar, Odisha 751013, India

**Keywords:** Protein scaffolds, Multi-enzyme complex, Binding modules, Dockerin-cohesin interactions, SpyTag-SpyCatcher system

## Abstract

The synthesis of complex molecules using multiple enzymes simultaneously in one reaction vessel has rapidly emerged as a new frontier in the field of bioprocess technology. However, operating different enzymes together in a single vessel limits their operational performance which needs to be addressed. With this respect, scaffolding proteins play an immense role in bringing different enzymes together in a specific manner. The scaffolding improves the catalytic performance, enzyme stability and provides an optimal micro-environment for biochemical reactions. This review describes the components of protein scaffolds, different ways of constructing a protein scaffold-based multi-enzyme complex, and their effects on enzyme kinetics. Moreover, different conjugation strategies viz; dockerin-cohesin interaction, SpyTag-SpyCatcher system, peptide linker-based ligation, affibody, and sortase-mediated ligation are discussed in detail. Various analytical and characterization tools that have enabled the development of these scaffolding strategies are also reviewed. Such mega-enzyme complexes promise wider applications in the field of biotechnology and bioengineering.

## Highlights

• Protein scaffold acts as a backbone with several interacting domains and ligands for the integration of specific enzymes.
• Different protein-enzyme ligation strategies are used for the co-localization of enzymes.
• Scaffolds permit control over spatial organization, enzyme stoichiometry, and proximity.
• Improvement in pathway flux and product yield can be achieved with scaffold-based multi-enzyme complex.
• Various analytical tools are utilized to study the multi-enzyme assembly process.

## 1 Introduction

Nature has always witnessed the highly organized enzymatic complexes that drive various metabolic reactions with a high degree of specificity. Indeed, in its highly ordered form, enzymes work more efficiently with better substrate channeling which subsequently enhances the overall productivity [1]. Such multi-enzyme clusters are often formed with the aid of scaffolds wherein enzymes assemble on desired docking sites on the scaffold biomolecule. Thus, the scaffolded clusters play an immense role in carrying out multi-step biochemical reactions. In recent decades, biocatalysis has been gaining considerable attention owing to its sustainable and environmentally friendly nature that in turn provides a greener alternative to traditional chemical synthesis. Tremendous efforts have been made in the field of biocatalyst engineering and biomimetics to design such multi-enzyme nanostructures resembling the naturally occurring scaffolded multi-enzyme complexes which would catalyze industrially relevant biochemical reactions with an enhanced rate of productivity. Several types of scaffold materials and technologies have been investigated to improve the overall processivity, stability, and substrate accessibility of enzymes along with the aim of increasing the enzyme loading capacity of scaffold materials. These improvements compensate for the high cost of enzyme biocatalyst in biotechnological industries.

Scaffolding material can be classified into two categories: synthetic scaffolds and natural macromolecular scaffolds (Table 1). In the context of synthetic scaffolds, diverse nanomaterials with a high aspect ratio (ratio of surface area to volume) have been explored to improve enzyme loading and thereby reducing the diffusional barrier. This strategy provides better conversion yield and improved catalytic activity [2]. To date, numerous versatile nanomaterials such as nanotubes [3], nanowires [4], nanoparticles [5], nanosponges [6], nanoflowers [7], metal-organic frameworks [8], nanocages [9], and nanocomposites [10] have been reported to be utilized for the construction of artificial scaffolds for enzyme cascades.

**Table 1:** Scaffold materials utilized for enzyme immobilization and their potential applications.

Scaffold type	Scaffold material	Enzymes immobilized	Application	Reference
Synthetic scaffolds	Polystyrene nanospheres	Endoglucanase	Biofuel production	[11]
	CeO_2_-TiO_2_ Nanocomposites	Lactate oxidase	Electrochemical biosensors	[10]
	Zirconium based Metal-Organic Frameworks	Cellulase	Biomass valorization	[12]
	Multi-walled carbon nanotube	Lipase	Synthesis of fruit flavors	[3]
	Chitosan magnetic nanoparticles	Pectinase	Clarification and stabilization of fruit juices	[13]
	SBA-15 mesoporous sieves	Acetylcholinesterase	Detection of organophosphorus and carbamate pesticide	[14]
Biological supramolecular scaffolds	Polyhydroxyalkanoates(PHA)	Organophosphorus anhydride hydrolase	Bioremediation	[15]
	Magnetosomes	*β*- glucuronidase	Biomedical application	[16]
	Bacteriophages P22 virus-like particles (VLPs)	Peroxygenase CYP_BM3_21B3 and Glucose oxidase	Biotransformation of endocrine disruptor compounds	[17]
	Forisomes	Glucose-6-phosphate dehydrogenase and Hexokinase 2	Biosensor and microfluidic devices	[18]
	Apoferritin	Human carbonic anhydrase, Retro-aldolase, and Kemp eliminase	Pharmaceutical and nanotechnological application	[19]
	RNA scaffold	(FeFe)-hydrogenase and ferredoxin	Hydrogen production	[20]
	DNA nanostructure	Glucose oxidase and horseradish peroxidase	Biosensor and biotechnological application	[21]

Natural macromolecular scaffolds have also been widely employed where nucleic acids and proteins are used as scaffold materials. For instance, DNA, as well as RNA in their one or two-dimensional geometries, organizes the enzyme cascades into a complex by using well-established nucleic acid-based methodologies [20, 22]. However nucleic acid-based nanoscaffolds often suffer from the high cost of synthesis along with the struggle of docking the enzymes on it without affecting their biocatalytic activity [23]. On the other hand, proteins can be an interesting alternative candidate for the localization of enzyme/s since they can be genetically modified, can be produced in large quantities in heterologous hosts, and can be conjugated with the enzyme/s by integration of simple molecular recognition domain [24]. Thus, a diverse range of proteins has been explored to develop a versatile scaffold system for achieving the desirable productivity of the metabolic pathway. In this review, we highlight various ways of enzyme assembly on a protein backbone, different molecular recognition strategies for protein-enzyme conjugation, and characterization of the whole complex. We then discuss diverse novel protein assemblies that have been exploited so far as a scaffolding platform for multi-enzyme assembly.

## 2 Protein scaffold system

In nature, scaffold proteins are involved in signaling cascades where they serve docking sites for various protein members of the signaling cascade thereby smoothing out corresponding interactions and functions [25]. Being genetically modifiable; such nanoscale protein-based carriers could efficiently organize complex enzyme cascades comprising two or more types of enzymes in specified configuration, subsequently improving the enzymatic performance along with the pathway flux [26]. Furthermore, the scaffold-based multi-enzyme complex also decreases the loss of intermediates due to proximity of catalytic sites, decreases overall transit time, and reduces product feedback inhibition [27, 28].

Cellulosome is one such distinctive example of a naturally occurring protein scaffold system comprising structural backbone (scaffoldins) where cellulases have been localized via dockerin-cohesin interactions [29]. Owing to better catalytic efficiency and highly organized structure, cellulosomes have potential applications in various biorefineries [30]. To date, various researchers have used the scaffoldin and interactive domains from natural cellulosomes for the colocalization of various enzymes. For instance, alcohol dehydrogenase (ADH), formate dehydrogenase, and formaldehyde dehydrogenase have been assembled on scaffoldin for NADH production whereas amidohydrolase and hydantoinase assembled for semi-synthetic antibiotic production [31, 32].

**Fig. 1:**
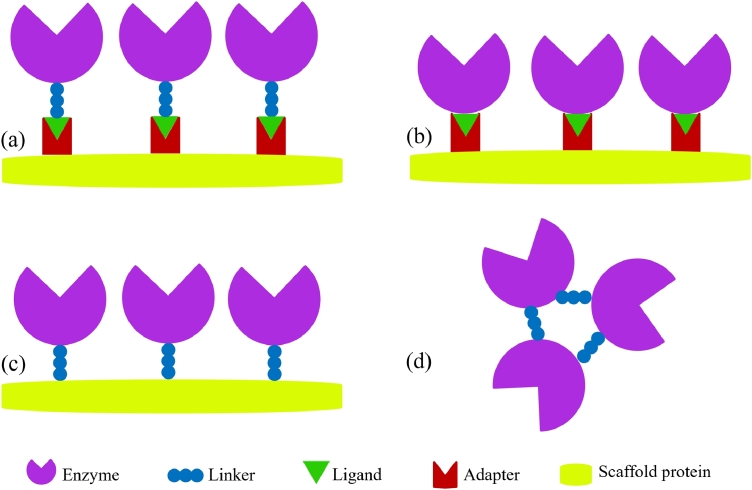
Enzyme scaffolding strategies; (a) Adapter domain-ligand mediated enzyme assembly on scaffold protein with peptide linker as a connecting bridge; (b) Direct assembly of enzymes on scaffold protein through adapter domain-ligand interactions; (c) Direct fusion of enzymes on scaffold protein via peptide linker; (d) Peptide linker based enzyme cross-linking.

Different strategies that are applied to design protein scaffold systems are depicted in Fig. 1. The basic structural units of protein scaffolds are adapter domain, peptide motifs/ligands, and linker. These three building blocks of the scaffold system affect the overall shape of the scaffold [33]. Adapter domains are small protein-binding modules of adapter proteins that permit specific protein-protein interactions in a highly regulated fashion. Phosphotyrosine binding domain, Src homology 2, and Src homology 3 domains are few well-known examples of adapter domains [34]. Peptide motifs or ligands are linear interactive peptides having short amino acid sequences complementary to adapter domains. Linkers act as connecting bridges between engineered enzymes and attached peptide motifs (Fig. 1a). Alternatively, peptide ligands can also be directly fused with enzymes eliminating the use of linkers for the development of scaffold systems (Fig. 1b). On the contrary, enzymes can be directly fused with scaffold proteins via linkers for the building of multi-enzyme complexes (Fig. 1c). Lastly, linkers can be directly used to interlink the multiple enzymes with each other to form multi-enzyme complexes (Fig. 1d). While selecting a particular method for enzyme scaffolding, one must consider the kind of enzyme/s (type/s as well as copy number) to be immobilized and their substrate-enzyme reaction/s as it may block active sites of enzyme/s and affect overall reaction kinetics respectively if the selected method is unsuitable for enzyme cascade.

## 3 Strategies of enzyme immobilization on a protein scaffold

**Fig. 2:**
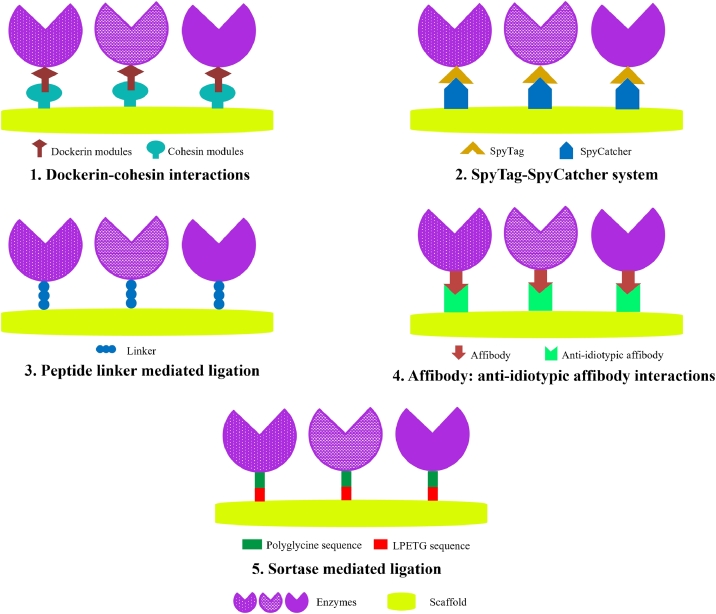
Overview of recent enzyme-protein ligation strategies.

There are two general ways of enzyme immobilization using protein nanocarrier viz; surface localization and encapsulation. In both cases, enzymes can be covalently or non-covalently associated with their scaffold protein. Following are some recent strategies applied for the ligation of enzymes to scaffold protein (Fig. 2).

### 3.1 Dockerin-cohesin interaction

Dockerin-cohesin interactions are a vital part of the cellulosome where cohesin modules are associated with scaffoldin and are responsible for organizing the cellulolytic enzymes. Whereas, dockerin bearing enzymes anchor to the scaffoldin via high-affinity dockerin-cohesin interactions having dissociation constant (K_d_) ranging from 10^−9^ M to 10^−12^ M [35, 36]. Ca^+2^ ions are essential for the interactions due to the presence of calcium-binding motif in the dockerin domain [37]. Karpol et al. have developed an affinity-based protein purification system using dockerin-cohesin interactions where cohesin module was immobilized on the beaded cellulose (affinity resin matrix) via carbohydrate-binding domain and targeted protein was tagged with truncated dockerin (affinity tag). The targeted protein binds to the column matrix through dockerin-cohesin interactions that later effectively eluted out using gradients of EDTA. Reutilization study further confirms the reusable nature of the affinity matrix for protein purification [38].

Dockerin-cohesin interactions were used to construct a cytosolic synthetic scaffold system in *Saccharomyces cerevisiae* for the production of 2,3-butanediol in another study. This increased the production titer by 37% [39]. Similarly, improvement of xylitol production was achieved by displaying xylose reductase (XR) and phosphite dehydrogenase (PTDH) on the outer coat protein (CotG) of *Bacillus subtilis* spores by controlling the XR/PTDH stoichiometry. XR and PTDH copy number on the spore surface was controlled by using a dockerin-cohesin module from two different sources viz; *Clostridium thermocellum* type 1 dockerin-cohesin module for XR and *Ruminococcus flavefecians* type 1 dockerin-cohesin module for PTDH. Furthermore, the stability of XR and PTDH was improved by 2.8-fold and 2.3-fold respectively at 25 °C after 10 h of incubation [40].

### 3.2 SpyTag-SpyCatcher system

The Howarth laboratory has developed the SpyTag-SpyCatcher domain system that is widely used for the colocalization of different enzymes [41, 42]. This system is formed by splitting the CnaB2 domain of the surface protein (FbaB) of *Streptococcus pyogenes*. SpyTag is a short, unfolded, versatile 13 amino acids long peptide sequence consisting of reactive aspartic acid residue which upon recognition of reactive lysine residue of its partner protein i.e. SpyCatcher, forms a covalent isopeptide bond [43]. The chemistry of the SpyTag-SpyCatcher bioconjugation process has been substantiated to be very rapid, highly efficient, independent of its position on the protein sequence, and highly robust in nature with stability at wide reaction conditions of temperature (4–37 °C), pH (4–8), and in the presence of various detergents (Tween-100, Tween-20, CHAPS, Nonidet P-40 except SDS) [44].

Jia et al. have constructed a polymeric SpyCatcher scaffold whose feasibility was initially inspected by conjugating SpyTagged enzymes (endoxylanase and arabinofuranosidase) on polymeric SpyCatcher construct in a site-specific and ratio-controllable manner and achieved 53% higher sugar conversion yield [45]. The construct was further explored for the detection of ovalbumin in ELISA by conjugation of SpyTagged Nanoluc and protein G on the scaffold [45]. Another research group has developed a single-step method for the purification and immobilization of xylanase-lichenase chimera [46]. SpyTag was fused between xylanase and lichenase to form a chimera that covalently binds to SpyCatcher-elastin like polypeptides (purification tag) via *in vitro* spontaneous SpyTag-SpyCatcher reaction and simultaneously self-assembled to form insoluble active particles during purification process which serves as immobilized enzyme complex. This immobilized enzyme chimera improved the stability by retaining 44% and 56% activities of xylanase and lichenase respectively even after 10 subsequent reaction cycles. However, a 1.7-fold (xylanase) and 1.1-fold (lichenase) decrease in the catalytic efficiency of immobilized enzyme was found [46].

Dovala et al. demonstrated SpyCatcher-SpyTag based rapid analysis of proteins where fluorophore tagged SpyCatcher binds to SpyTagged target proteins from cell lysate by SpyCatcher-SpyTag interactions during the pre-incubation period before electrophoresis [47]. Direct fluorescence imaging of gel following electrophoretic protein separation gives highly specific western blot-like information with the least reagents. Furthermore, this fluorophore-SpyCatcher was effectively used to analyze mono-dispersity, expression level, aggregation state, and solubility of tagged protein using fluorescence size exclusion chromatography before any purification [47]. Overall, these examples prove the utility of the versatile, robust SpyCatcher-SpyTag system in biotechnological and analytical fields.

### 3.3 Peptide linker mediated ligation

Two or more enzymes are genetically fused through short peptide bridges to create scaffolded multi-enzyme cascades. Such short peptide linkers could effectively facilitate better substrate channeling between the fused enzymes [48]. Haga et al. demonstrated the effect of linker on the monooxygenase activity of proliferating cell nuclear antigen (PCNA)-based multi-enzyme complex comprising of *Pseudomonas putida* cytochome P450 (P450cam), putidaredoxin reductase (PdR), and putidaredoxin (PdX). In this study, authors examined the effect of the poly-l-proline-rich linker between PCNA subunit 2 and PdX and reported 1.9 fold improvement in the overall monooxygenase activity [49]. Furthermore, the effect of flexible (Glycine)_4_-(Serine) repeating linker on the monooxygenase activity was explored with no change in the individual activities [49]. In another example, the effect of linker configuration on the structure and activity of formate dehydrogenase and leucine dehydrogenase in terms of linker type (rigid or flexible) and copy number was exploited and increased enzyme activity and thermal stability of fused enzymes were obtained with rigid peptide linker than flexible linker [50]. Both the findings suggest that rigid peptide linkers are more appropriate for improving catalytic performance and to fine-tune spatial arrangement of enzymes in multi-enzyme complex [49, 50]. Linkers have been widely used for improving the direct channeling of intermediates between catalytic modules of polyketide synthase [51, 52]. Also, this approach has been successfully implemented for the synthesis of bifunctional enzymes mainly for cost-effective recycling of co-factors [53, 54, 50].

### 3.4 Affibodies

Affibodies are relatively smaller (6 kDa) and less complex immunoglobulin-like affinity proteins consisting of 58 non-cysteine residues of three-helix bundle domains derived from the Z domain of protein A from *Staphylococcus aureus* [55]*.* The phage display technique is used for the production of randomized phage display affibody libraries to create ligand binding variants from which high-affinity ligand binding affibody is selected after bio-panning [56, 57]. These affibody molecules are known to have fast-folding kinetics with extreme pH and temperature stability that make them a favorable tool for scaffolding enzyme cascades. For scaffolding multiple enzymes, affibody:anti-idiotypic affibody binding pairs are used where anti-idiotypic affibodies are raised against affibodies to form high-affinity interaction with K_d_ value of 0.05 μM to 0.9 μM [58, 59]. Eklund et al. have designed staphylococcal protein A based scaffold that mimics the architecture of cellulosomes where engineered affibody:Ig-binding domain interactions replace dockerin-cohesin interactions. This designer cellulosome, when fused with carbohydrate-binding domain, binds efficiently to the cellulose surface. However, enzyme assembly on designer cellulosome and its effect on enzyme kinetics were not examined under this study [60].

Few reports are available stating colocalization of enzymes using affibody:anti-idiotypic affibody pairs on synthetic scaffold proteins. In one such study, a bifunctional enzyme scaffold was formed by linking two different anti-idiotypic affibodies (anti-Z_Taq_ and anti-Z_IgA_) on which affibody fused enzymes (Z_Taq_ fused farnesene synthase and Z_IgA_ fused farnesyl diphosphate synthase) were localized *in vivo* by affibody:anti-idiotypic affibody interactions in *S. cerevisiae* and 135% increase in yield of farnesene was achieved [59]. This strategy was further extended to three enzyme cascades of the poly-hydroxybutyrate pathway in *Escherichia coli* and resulted in a seven-fold increase in poly-hydroxybutyrate yield [59]. This finding further potentiates the use of affibodies in the field of metabolic engineering.

### 3.5 Sortase mediated ligation

Enzyme-mediated protein-protein ligation is another popular strategy of bioconjugation of enzymes to the protein scaffold structure. Sortase A is one such enzyme that assists isopeptide bond formation between the proteins of interest. Sortase A is membrane-bound cysteine transpeptidase expressed by *Staphylococcus aureus* [61]. In the presence of Ca^+2^, sortase A first recognizes its substrate *i.e.* peptide motif LPXTG, normally fused with protein to be ligated at its C-terminal region. Immediately upon recognition, it cleaves the LPXTG peptide sequence between the peptide bond of threonine and glycine leading to the formation of thioester intermediate. Afterward, another nucleophilic substrate of sortase A *i.e.* N- terminal oligo glycine peptide tag of the second target protein reacts with thioester intermediate by nucleophilic attack leading to the ligation of the target proteins by isopeptide bond formation thereby liberating ligated proteins and free sortase A enzyme [62]. Though the entire process seems to be convenient for scaffolding enzyme cascade, the low binding affinity (K_m_ value >5.0 mM), poor reaction kinetics extending reaction time, and lowering ligation efficiency constrain the widespread adoption of this strategy [63].

Utilizing a high molar concentration of enzyme as well as nucleophilic peptide substrate could enhance the ligation efficiency but at the expense of high cost. The consequences of such an approach may not be economically feasible for constructing scaffold systems for industrial applications. However, advancement in protein engineering has surpassed the limitations of sortase A [64, 65, 66]. Alternatively, a proximity-based sortase A mediated ligation approach with 95% ligation efficiency can be used for bioconjugation [67]. Sakamoto et al. attempted to ligate luciferase, alkaline phosphatase, and glucose oxidase (GOx) on the ZZ domain using sortase A with no significant effect on the enzyme activities [68]. Whereas, metabolic channeling of pyruvate formate lyase and phosphate acetyltransferase through sortase A mediated ligation diverts the central metabolic flux towards the acetate in the cytoplasm of *E. coli* [69]. Similarly, McConnell and co-workers have designed nanocage (T33–21) as a scaffold to exploit cellulase synergy utilizing colocalization of Cel48S exoglucanase and Cel8A endoglucanase from *C. thermocellum*, where enzymes were tagged with short polyglycine peptide whereas, LPXTG sequence was fused with scaffold backbone for sortase A mediated multi-enzyme assembly. The increase in activity (2.7-fold) was obtained with cage:Cel48S/Cel8A than the mixture of singly modified cages (cage:Cel48S and cage:Cel8A) [70].

## 4 Protein scaffolds employed in multi-enzyme assembly

There are numerous proteins present in nature that fascinatingly self-assemble into different nanostructures; mainly via protein-protein interactions. Due to current advancements in protein engineering, the self-assembly process could be controlled to create specifically designed protein scaffolds into precise supramolecular structures such as nanocages, filaments, rings, crystals, tubules, etc. [71]. From the last decade, researchers have designed various advanced nanobiocatalyst based on self-assembled protein scaffolds as a way to improve the stability and productivity of enzymes. Such protein nanocarriers and their effect on the kinetic parameters of the enzyme/s being immobilized are given in Table 2. In the following section, such nanobiocatalysts will be elaborated as per the type of protein scaffold used to either encapsulate enzymes in their interior or to display it on their exterior.

**Table 2:** Kinetic analysis of multi-enzyme assembly.

**Protein carrier**	**Enzyme**	**Immobilization strategy**	**Kinetic parameters**						**Reference**
			**V_max_**		**K_m_**		**k_cat_**		
			**Free enzyme**	**Immobilised enzyme**	**Free enzyme**	**Immobilised enzyme**	**Free enzyme**	**Immobilised enzyme**	
Elastin-like polypeptide	MenD - enzyme involved in menaquinone biosynthesis.	SpyCatcher-SpyTag mediated covalent bonding	∼2.7 μM.min^−1^	Cyclic assembly: 5.0 μM.min^−1^; Cross-linked assembly: 3.5 μM.min^−1^	32.9 ± 1.7 μM	Cyclic assembly: 32.9 ± 3.0 μM; Cross-linked assembly: 15.40 ± 1.6 μM	1.45 ± 0.03 min^−1^	Cyclic assembly: 2.57 ± 0.04 min^−1^; Cross-linked assembly: 1.60 ± 0.04 min^−1^	[72]
Ferritin	α-amylase	EDC/NHS mediated cross-linking	10.6 × 10^−5^ U.mg^−1^	3.3 × 10^−5^ U.mg^−1^	2.63 mg.mL^−1^	5.19 mg.ml^−1^	-	-	[73]
Apoferritin	Glucose oxidase	Streptavidin- biotin based non-covalent assembly	0.05 mM.min^−1^.mg^−1^	0.51 mM.min^−1^.mg^−1^	9.95 mM.L^−1^	7.54 mM.L^−1^	Retained activity at 50°C:		[74]
							50% for 1.5 h and 20% for 3 h	90% for 1.5 h and 50% for 3 h	
T4 phage capsid	Hoc fused amylase, maltase, and glucokinase	SpyCatcher-SpyTag mediated covalent bonding	Hoc-enzyme fusion mix: 6.31 ± 0.12 nM.s^−1^; Free enzyme mix: 4.33 ± 1.21 nM.s^−1^	78.50 ± 4.9 nM.s^−1^	Hoc-enzyme fusion mix: (3.00 ± 0.71) × 10⁵ nM; Free enzyme mix: (3.77 ± 1.59) × 10⁴ nM	(2.50 ± 0.20) × 10⁶ nM	Hoc-enzyme fusion mix: 0.32 ± 0.01 s^−1^; Free enzyme mix: 0.87 ± 0.24 s^−1^	3.93 ± 0.25 s^−1^	[75]
Human ferritin H chain	β-glucosidase	*E. coli* K coil and E coil interactions	k_cat_/K_m_ value:		1.26 mM	1.44 mM	65.26 s^−1^	70.55 s^−1^	[26]
			51.79 s^−1^.mM^−1^	48.99 s^−1^.mM^−1^					
Twigged streptavidin polymer scaffold	Cellulase	Sortase A-based ligation	-	-	-	-	Amount of reducing sugar released:		[61]
							On PSC: 1.60 g.L^−1^; On avicel: 0.16 g.L^−1^	On PSC: 2.00 g.L^−1^; On Avicel: 0.20 g.L^−1^	
Synthetic protein	Triosephosphate isomerase, aldolase and fructose 1,6-bisphosphatase	Cohesin-dockerin based high affinity non-covalent interaction	k_cat_/K_m_ value:		Enzyme mixture: 1.63 ± 0.28 mM	0.46 ± 0.12 mM	Enzyme mixture: 3.90 ± 0.29 min^−1^	36.30 ± 2.90 min^−1^	[76]
			Enzyme mixture: 2.39 mM^−1^.min^−1^	79.70 mM^−1^.min^−1^					
Synthetic protein	Lipase and p450 fatty acid decarboxylase	Cohesin -dockerin based high affinity non-covalent interaction	k_cat_/K_m_ value:		-	-	Enzyme productivity:		[77]
			Enzyme mixture: 2.10 × 10⁴ M^−1^s^−1^	1.30 × 10⁶ M^−1^s^−1^			Enzyme mixture: 0.17 µM.min^−1^	4.60 µM.min^−1^	
Interacting proteins- IPA/IPa, IPB/IPb, and IPC/IPc	Endoglucanase (EG), exoglucanase (CBH) and β- glucosidase (BGL)	Glycine-serine linker mediated covalent bonding	-	-	Enzyme activity:		Specific activity:		[78]
					After a 4-h reaction, a 1.5-fold increase in enzyme activity for the tri-enzyme complex than corresponding free enzymes		BGL: ∼1.5 U.μM^−1^; CBH: ∼ 1.60 U.μM^−1^; EG: ∼1100 U.μM^−1^	BGL: ∼1.75 U.μM^−1^; CBH: ∼ 1.70 U.μM^−1^; EG: ∼ 820 U.μM^−1^	
SP1	Cellulase	Cohesin(Coh)-Dockerin (Doc) based high affinity non-covalent interaction	A cellulase constructs having a longer linker to its dockerin, termed Doc-l-cellulase.				Specific activity:		[79]
							Doc-cellulase: 9.0 × 10² U.μM^−1^; Doc-L-cellulase: 4.3 × 10² U.μM^−1^	Coh-SP1 + Doc-cellulase: 15.0 × 10² U.μM^−1^; Coh-SP1 + Doc-L-cellulase: 8.5 × 10² U.μM^−1^	
EutM	Alcohol dehydrogenase (ADH)	SpyCatcher−SpyTag mediated covalent bonding	-	-	-	-	Specific activity:		[42]
							ADH: 1100 mU.mg^−1^; SpyTag-ADH: 1500 mU. mg^−1^	1.6 fold higher than SpyTag-ADH	
Gamma-prefoldin (γ-PFD)	Glucose oxidase (GOx) and horseradish peroxidase (HRP)	SpyCatcher−SpyTag mediated covalent bonding	-	-	HRP: 0.9 ± 0.2 × 10^−^³ M; SpyCatcher-HRP: 1.0 ± 0.2 × 10^−^³ M; GOx: 21 ± 4 × 10^−^³ M; SpyCatcher-GOx: 21 ± 2 × 10^−^³ M	SpyCatcher-HRP + γ-PFD scaffold: 1.1 ± 0.2 × 10^−^³ M; SpyCatcher-GOx + γ-PFD scaffold: 18 + 2 × 10^−^³ M	HRP: 2900 ± 800 s^−1^; SpyCatcher-HRP: 2400 ± 500 s^−1^; GOx: 160 ± 30 s^−1^; SpyCatcher-GOx: 150 ± 20 s^−1^	SpyCatcher-HRP + γ-PFD scaffold: 4700 ± 900 s^−1^; SpyCatcher-GOx + γ-PFD scaffold: 210 ± 20 s^−1^	[80]
Apoferritin (AfFtn)	GFP fused enzymes: human carbonic anhydrase (G-CA), (retro-) aldolase (RA-G) and Kemp eliminase (G-KE)	Encapsulation	k_cat_/K_m_ value:		RA-G: 300 ± 20 µM; G-KE: 1700 ± 200 µM	RA-G+ AfFtn: 280 ± 30 µM; G-KE+ AfFtn: 1400 ± 100 µM	RA-G: 4.3 ± 0.1 s^−1^; G-KE: 170 ± 10 s^−1^	RA-G+ AfFtn: 6.2 ± 0.4 s^−1^; G-KE+ AfFtn: 150 ± 30 s^−1^	[19]
			G-CA: (1.4 ± 0.4) × 10³ M^−1^s^−1^; RA-G: (1.4 ± 0.2) × 10⁴ M^−1^s^−1^; G-KE: (9.9 ± 1.0) × 10⁴ M^−1^s^−1^	G-CA+ AfFtn: (1.2 ± 0.3) × 10³ M^−1^s^−1^; RA-G+ AfFtn: (2.2 ± 0.2) × 10⁴ M^−1^s^−1^; G-KE+ AfFtn: (11.2 ± 2.5) × 10⁴ M^−1^s^−1^					

### 4.1 Virus-like particles

Virus-like particles (VLPs) are non-infectious, self-assembling nanocages derived from discrete numbers of viral capsid proteins that resemble the overall structure of virus particles but are devoid of native viral machinery [81]. These VLPs are widely used as a nanoreactor where enzymes are encapsulated within the interior of VLPs. Encapsulation of enzymes within VLPs is a promising approach to mitigate enzyme degradation by protease attack, pH shifts, and high-temperature conditions. The ability to control the pore sizes of VLPs further improves the selectivity of enzymes as it facilitates selective entry and exit of substrates and products. Another benefit of using VLPs is the compartmentalization of complex multi-enzyme cascades intended to simulate the multi-enzyme micro-compartments found in nature; for example, ethanolamine utilization micro-compartments [82]. Following are some examples of VLPs designed for enzyme encapsulation.

#### 4.1.1 Cowpea chlorotic mottle virus

Cowpea chlorotic mottle virus (CCMV) is a 28 nm icosahedral single-stranded RNA plant virus that belongs to the family of *Bromoviridae*. It is made up of 180 identical copies of coat proteins (CP) with T3 triangulation number that self-assembled into 20 hexamers and 12 pentamers. One of the appealing properties of CCMV is, an *in vitro* reversible self-assembly of CP. Additionally, the CCMV capsid shell is known to contain multiple pores of roughly 2 nm in size which allow diffusion [83]. These features make CCMV a suitable candidate for the production of a CCMV VLP based nanoreactor. With regards to this, CCMV capsid disassembles into dimers to release its RNA cargo at pH ≥ 7.5 and under higher ionic strength (∼1 M). Consequently, RNA is removed by precipitation using calcium ions, and resultant CP dimers are re-assembled to form CCMV VLPs by lowering the pH towards 4.5 [84, 82].

Various strategies have been applied for the encapsulation of enzymes inside the CCMV VLPs. One study utilized the pH-dependent responsiveness of CCMV capsid to incorporate horseradish peroxidase within the capsid [85]. In another study, two different enzyme pairs viz, GOx:DNAzymes and GOx:gluconokinase were encapsulated via non-covalent electrostatic interactions between negatively charged single-stranded DNA tagged enzymes and positively charged interior of capsid during the assembly process [83]. Furthermore, the Sortase A-mediated ligation strategy has also been used to demonstrate highly efficient cargo loading in CCMV VLPs wherein the polyglycine peptide is fused with N-terminal of CP and LPETG tag was fused to target protein followed by sortase A mediated isopeptide bond formation between them [86].

#### 4.1.2 P22

P22 bacteriophage is a temperate phage of *Salmonella typhimurium* that belongs to the *Podoviridae* family. The P22 capsid structure exhibit T7 triangulation number icosahedral symmetry consisting of 420 copies of 46.6 kDa CP that self assembles on to the approximately 100–330 copies of 33.6 kDa scaffolding protein (SP) through non-covalent interactions with C-terminus of SPs forming compact procapsid (PC) structure [87]. The PC structure is (58 nm diameter) double in size of CCMV VLPs and can form different capsid architecture with alterations in capsid porosity and internal volume by simply changing the incubation temperature and time of capsid formation. Heating at 60 °C for 15 min irreversibly changes the PC form to an expanded shell form (EX) with a doubling of the internal volume and increase in the diameter (60 nm) but with the loss of SPs. These two forms can be further transformed into wiffleball form (WB) by heating at 70 °C for 20 min. During the heating process, CP pentons dissociate from each of the five vertices in the EX capsid structure which leads to the formation of 10 nm of pores in the resulting WB VLPs with no change in the diameter [88]. All three morphologies of P22 VLPs allow researchers to easily modulate the porosity and internal volume of capsid to design a versatile nanoreactor.

Directed encapsulation of enzyme cascade involved in sugar metabolism within a P22 VLPs was reported which comprises *β*-glucosidase (CelB), ATP-dependent galactokinase (GALK), and ADP-dependent glucokinase (GLUK) [89]. This was achieved by constructing multi-enzyme fusion where two and three enzymes fused with each other and to the SP monomers through a polyglycine flexible linker, thus forming CelB-GLUK-SP and GALK-GLUK-CelB-SP complex respectively. SP monomer directs the entry of multi-enzyme fusions inside the P22 VLPs. In both these cases, enzyme kinetics of resulting CelB-GLUK-P22 and GALK-GLUK-CELB-P22 VLPs was improved significantly [89]. Besides this, another study of green fluorescent protein (GFP) and head domain of hemagglutinin protein from influenza (HAhead) was attempted to display on the exterior surface of CP of P22 VLPs via a sortase based ligation approach [88]. Results support an overall concept and modularity of the approach where LPTEG tagged P22 VLPs scaffold can be used for the display of multiple proteins of interest [88].

The catalytic activity of encapsulated ADH was finely tuned by controlling the stoichiometry of enzyme loading and packing density of enzymes inside the P22 VLPs [90]. Compositional control was achieved by mixing different ratios of ADH fused SP (ADH-SP) and wild type SPs (wtSPs) during *in vitro* assembly of P22 VLPs whereas packing density was controlled by selective removal of wtSPs through P22 capsid pores using mild treatment with a chaotrope. The wtSPs were observed to exert a crowding effect on the enzyme which finely tuned the enzymatic output [90]. A similar approach was used to synthesize complex proteinaceous hierarchical structures with P22 VLPs that mimic cellular environments having different macromolecules and subcellular compartments [91]. As a proof of concept, the ferritin nanocage which acts as a separate confined compartment and *β*-glycosidase was co-encapsulated with a controlled stoichiometry of loading within P22 VLPs by fusion of the respective genes with SPs [91]. Altogether, the understanding gained from both the research could be useful to construct cell-like bioreactor for various biochemical reactions.

### 4.2 Ferritin

Ferritin is an iron storage protein present in all domains of a life consisting of 24 identical monomers that self-assemble into a cage-like structure with 12 nm of outer diameter and 8 nm of inner diameter [92, 74]. Ferritin can store up to 4500 Fe(III) atoms having paramagnetic properties which aid in easy recovery and reusability of scaffold system by an external magnetic field [73, 42]. It has wide pH and temperature stability. The pH-induced disassembly-reassembly process of apoferritin (ferritin cage without Fe(III) atoms) is pseudoreversible between a pH range of 10.00 to 2.66 [93]. Moreover, they are stable in various denaturants like sodium hydroxide, urea, and guanidinium chloride because of the presence of large numbers of salt bridges and hydrogen bonding between subunits [94]. Due to these marvelous properties ferritin has been used as a scaffold mainly in two different forms, viz; apoferritin and magnetoferritin.

Apoferritin has been used for the localization of enzymes such as GOx, aldolase, and α-amylase [19, 73, 95]. Improved enzyme kinetics with a ten-fold increase in V_max_ and a 24% decrease in K_m_ values was observed when GOx was displayed on the apoferritin via streptavidin-biotin interactions [95]. Furthermore, the stability of GOx was improved upon immobilization with a 1.8-fold and 2.5-fold increase in its retained activity for 1.5 h and 3 h respectively at 50 °C than that of free GOx. This immobilized GOx was also stable at high (5 M) urea concentration with negligible loss of activity wherein the free GOx lost around 80% of its activity [95]. In another study, human carbonic anhydrase (G-CA), (retro-)aldolase (RA-G), and Kemp eliminase (G-KE) were encapsulated within an apoferritin cage individually via electrostatic interactions. This improved enzyme kinetics but the enzyme loading was very low (only 2–3 enzyme molecules per cage) [19]. This finding is consistent with the fact that apoferritin has a very tiny nanocage of 8 nm diameter which cannot accommodate more enzyme molecules; thus limiting the use of enzyme encapsulation strategy for the construction of ferritin-based nanobiocatalyst.

Yu Zhang et al. have used the magnetic properties of ferritin protein to construct human H chain magnetoferritin based *β*-glucosidase enzyme complex that can be easily recovered from the reaction medium with an application of external magnetic fields [42]. This is a very promising approach that needs to explore because of its immense application in the development of robust recyclable industrial biocatalyst.

### 4.3 SP1 protein

Stable protein 1 (SP1) isolated from *Populus tremula* aspen plant is a 148.8 kDa ring-like homo-dodecameric protein structure with an outer diameter of 11 nm and an inner core of 3 nm. This self-assembled protein is composed of 12 subunits that are held together via hydrophobic interactions and are expressed during adverse environmental stress such as cold, salinity, and heat stress in the plant [79]. It is noteworthy that SP1 protein is known to exert remarkable stability in boiling temperature with a melting temperature of 107 °C along with resistance to protease attack (trypsin, proteinase K and V8), organic solvents, and ionic detergent [96, 97]. SP1 protein has been engineered to construct artificial cellulosome where cohesin module was fused with SP1 monomer thus forming a Coh-SP1 fusion while dockerin module was linked to cellulase with and without linker peptide termed Doc-l-cellulase and Doc-cellulase respectively [79]. A long 23-residue linker was expected to prohibit a potential steric hindrance thereby ensuring proper folding and assembly of Coh-SP1 and dockerin bearing cellulase via cohesin-dockerin interactions. Though integration of peptide linker between cellulase and dockerin module enhances an enzyme loading capacity of Coh-SP1 scaffold, a two-fold decrease in specific activity of Doc-l-cellulase and their resultant scaffold complex was observed [79]. In another study, this Coh-SP1 protein scaffold was used to display exoglucanase where the synergistic effect of scaffolded exoglucanase and free endoglucanase was investigated using cellulose substrate. This combination was found to elevate the overall hydrolysis of cellulose by 20% [98].

### 4.4 Shell protein of bacterial micro-compartments

Bacterial micro-compartments (BMC) are self-assembling semipermeable multi-component protein compartments or organelle with a size in a range of 40–600 nm found in bacteria. It encapsulates a variety of enzyme cascades involved in anabolic (eg. carboxysome) or catabolic metabolism (eg. ethanolamine utilization BMC, propanediol utilization BMC) where protein shell provides an interface with cytosol [99]. The shell of BMC is made up of three types of structural proteins, each having one or two domain sequences of either Pfam00936 (that form cyclic homo-hexamers or pseudo-hexamers) or Pfam03319 (that form cyclic homopentamers) to function as hexagonal facets and pentagonal vertices of the BMC structure [100, 99].

The self-assembling nature of the shell proteins further potentiates the bottom-up bioengineering perspective to develop the synthetic BMC nanofactories which could encapsulate diverse compounds viz; non-endogenous cargo proteins, drug compounds, and enzyme cascades [101]. For instance, 1,2- propanediol (POD) utilization BMCs from *Salmonella enterica* was heterologously expressed in *E. coli* with the co-expression of *β*-galactosidase, esterase, and cofactor dependent glycerol dehydrogenase. These enzymes were fused with encapsulation peptide via a flexible glycine-rich linker for *in vivo* directed assembly within BMCs. Notably, the enzyme activity of all three enzymes was retained significantly whereas additional protection against acidic pH was observed due to BMC shell [101]. However, the use of encapsulation peptides often leads to aggregation of enzymes and hence has low encapsulation efficiency. This issue has been circumvented by utilizing more specific strategies of enzyme-protein conjugation which would allow both *in vivo* and *in vitro* assembly of enzymes or cargo covalently on the surface of BMCs or within the core of BMCs [102, 103, 104, 42].

### 4.5 PFD filament protein

Prefoldins (PFD) are the family of molecular chaperones found in both archaea and eukaryotes where they assist accurate protein folding in an ATP-independent manner. It is a hetero-hexameric complex consisting of six different subunits (in eukaryotes) and two α and four *β* subunits (in archaea). For instance, gamma-prefoldin (γ-PFD), a filamentous chaperone protein isolated from *Methanocaldococcus janaschii* archean found in deep-sea hydrothermal vents. This γ-PFD can self-assemble by forming a dimer of monomers followed by subsequent oligomerization of dimers through *β* bundle formation thus forming a filamentous structure of average length around 127 nm [105]. This filamentous chaperone exhibits high thermal stability with a melting temperature (Tm) of 93 °C and stabilizes the other proteins from denaturation under unfavorable conditions [106]. All these features make them an ideal candidate for the controlled assembly of enzyme cascades.

Lim et al. have developed a versatile γ-PFD based scaffold system using SpyCatcher-SpyTag interaction domains that could scaffold a variety of enzymes or proteins of interest. The controlled immobilization of proteins on the scaffold was demonstrated using mCerulean3 and mVenus fluorescent protein. The impact of scaffolding on the catalytic activity was analyzed using horseradish peroxidase (HP) and GOx. The kinetic analysis revealed that the K_cat_ values of GOx-SpyCatcher and HP-SpyCatcher markedly increased upon localization on γ-PFD-SpyTag. On the contrary to this, colocalization of both the enzymes together on the γ-PFD-SpyTag scaffold did not further enhance sequential reaction, indicating no significant channeling of intermediates between the enzymes [80].

### 4.6 Casein

Casein is a type of phosphoprotein consisting of four types of peptides: αS1, αS2, *β*- and κ-casein (in case of bovine milk) that differs in amino acid composition but have similar amphiphilic nature. These mixed, as well as pure forms of peptide, can self-assemble into micelles of around 50–500 nm in an aqueous solution. This self-assembly is pH and temperature-driven thus making it possible to encapsulate a variety of active molecules [107]. Furthermore, transglutaminase-aided cross-linking of casein with protein of interest forms a biopolymer that is applied for enzyme assembly [108].

There is only one study of this application where an artificial cellulosome was constructed using *β*-casein and N,N-dimethyl casein (DM-casein) as a scaffold [109]. Casein consists of 20 glutamine (Q) and 12 lysine (K) residues which are highly reactive substrates for microbial transglutaminase (MTG). MTG catalyzes acyl transfer reaction where γ-carboxamide groups of Q residues in the casein protein acts as the acyl donor while ϵ-amino groups of K residues act as the acyl acceptor. This leads to the formation of ϵ-(γ-glutamyl)-lysine cross-linking within the protein of interest [110]. Therefore, this strategy has been utilized for the casein-enzyme conjugation where lysine-rich peptide sequence was fused with C-terminus of endoglucanase EG(Cel5A) and EG(Cel6A). Lysine residues of EG(Cel5A) and EG(Cel6A) undergo MTG mediated cross-linking with glutamine residues of *β*-casein and DM-casein forming casein-EG conjugate. *β*-casein also undergoes MTG-catalyzed self-cross-linking due to the presence of intrinsic reactive lysine residues but this could hinder the processivity of the enzyme in the conjugate [109]. Though, DM-casein couldn't undergo self cross-linking by MTG due to the presence of modified lysine residues; it can facilitate control over enzyme loading per DM-casein. Cellulose saccharification with EG-DM-casein conjugate showed a two-fold increase as compared to EG-*β*-casein conjugate enzyme: scaffold ratios were 1:6 [109]. The casein-based enzyme conjugates can be easily recovered from the reaction mixture by calcium-mediated precipitation. Hence, there is a great potential to explore and utilize the property of casein to improve the processivity of enzymes.

### 4.7 Amyloid like nanofibrils

Amyloid nanofibrils are straight, unbranched, ubiquitous peptide/ protein fibrous structures that are formed by the nucleation process. It is initiated with the self-assembly of soluble amyloidogenic peptides or proteins to form protofibrils onto which several other amyloidogenic peptides or proteins aggregate in highly ordered *β* sheet structures giving rise to a mature amyloid fiber [111]. Amyloid fibrils are generally 6–12 nm in diameter and few micrometers in length. These amyloid nanofibrils exist in two forms viz; nontoxic form essential for biological functions and disease-associated toxic form [111, 112].

There are few reports available on scaffolding the enzymatic cascades on nontoxic protein nanofibrils [113, 114]. One such example is where Sup35 amyloidogenic peptides were fused with xylanase A (XylA), *β*-xylosidase II (β-xyl), and aldose sugar dehydrogenase (ASD) to form three enzymatically functionalized fibrils for the production of xylonolactone [114]. The catalytic activity of enzymes was found to be unaffected in single enzyme-containing fibrils. Since there are differences in pH optima of the hemicellulases (pH 6.5) (XylA and β-xyl) and ASD pH (9.0), an 11-fold decrease in yield was obtained from the biocatalytic reaction at pH 7.5. To circumvent this issue, the author applied another strategy where xylose produced from XylA/*β*-xyl fibril complex was directly used as a substrate for ASD fibril complex at pH 9.0. This strategy was found to be more productive than immobilizing all three enzymes together on fibrils [114].

## 5 Characterization of protein scaffold-based enzyme assembly

**Fig. 3:**
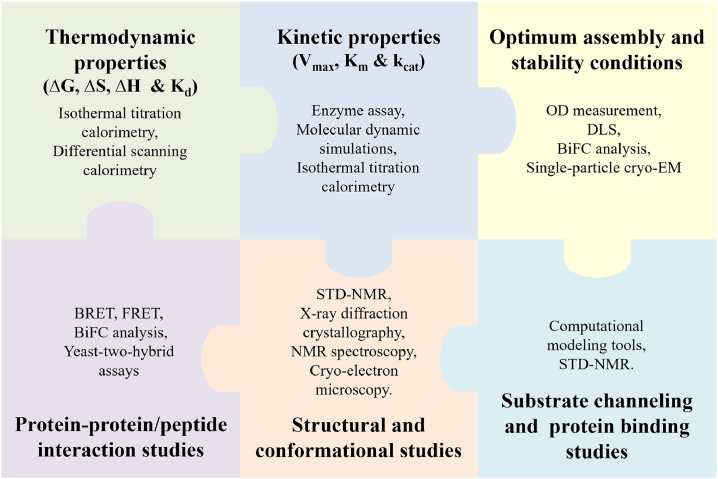
Characterization Techniques for multi-enzyme complexes. (BiFC- bimolecular fluorescence complementation; BRET- bioluminescence resonance energy transfer analysis; FRET- fluorescence resonance energy transfer analysis; NMR- nuclear magnetic resonance; STD-NMR- saturation transfer difference NMR; cryo-EM- cryogenic electron microscopy).

The strategies for the multi-enzyme assembly such as direct fusions, cross-linking and scaffold mediated assembly, may suffer from non-specific interactions during *in vivo*/*in vitro* assembly process which affects the overall processivity and stability of assembled enzymes. Hence, a study of enzyme binding parameters is crucial for understanding the entire process of multi-enzyme assembly. For this, exploring thermodynamic as well as kinetic properties of the multi-enzyme complex is of utmost importance (Fig. 3). An isothermal titration calorimetry is a major tool for thermodynamic characterization that investigates the types of interactions (hydrophobic, hydrophilic, electrostatic, polar, or non-polar) between the enzyme and scaffold. This is achieved by calculating the enthalpy (∆H), entropy (∆S), Gibbs free energy (∆G), reaction stoichiometry (n), and binding constant (K_D_) of the reaction simply by measuring the heat absorbed or evolved during the binding process [115]. Yet another tool named differential scanning calorimetry (DSC), a non-perturbing technique that can measure the molar heat capacity, phase transitions, conformational changes, and enthalpy changes. Molar heat capacity is directly used to investigate all the thermodynamic properties [116]. DSC-based thermal protein denaturation study provides information about the forces involved in the protein's conformational stability as well as the protein's unfolding process. The protein folding process can also be analyzed by measuring thermotropic changes in different reaction states [117]. It has been found that apart from enzymatic assay, enzyme kinetics can also be investigated using isothermal titration calorimetry [118, 119].

However, several authors have used optical density measurement (OD_600_) as the simplest approach to characterize turbidity which is indicative of the multi-enzyme complex formation under different reaction conditions [120, 121]. Dynamic light scattering (DLS), a non-invasive technique has been used for the determination of the particle size of the protein complexes which are in the range of 0.001 µm to several microns. The thermal stability of the protein complex can also be assessed by using this tool as proteins denature under the influence of heat causing massive aggregation of denatured proteins which subsequently results in a significant increase in size and scattering intensity [122]. Similarly, protein stability in different pH and concentrations can also be explored using DLS [123]. It has also been used for detecting assembly & disassembly processes of supramolecular structure [124, 120]. Furthermore, the overall binding mechanism, kinetics of the assembly process, and the conformational changes that occur during interactions can be studied using a molecular dynamics simulation approach [125, 126].

The protein-protein interactions during the assembly process can also be explored *in vivo* using conventional methods such as immunoprecipitation assay, bioluminescence resonance energy transfer (BRET) analysis, fluorescence resonance energy transfer (FRET) analysis, bimolecular fluorescence complementation (BiFC) analysis, and yeast-two-hybrid assays [127]. Recently, BiFC analysis is more widely accepted due to its advantages over other methods. BiFC analysis has been reported to study *in vivo* target protein interactions and their localization. Moreover, it is also been used to monitor the assembly-disassembly process of multi-enzyme complexes [128, 120].

Furthermore, the structural and functional analyses of multi-enzyme complexes are also crucial for investigating the processivity of enzyme components of the system as well as to get better mechanistic insights. Saturation transfer difference-nuclear magnetic resonance study (STD-NMR) has been reported to be used for identifying potential binding hotspots on the protein scaffold where different molecules can bind [129]. X-ray diffraction crystallography is a well-established method for structural analysis of proteins at atomic resolution [130]. Besides NMR and X-ray crystallography, for the past two decades, Single-particle Cryo-electron microscopy is gradually gaining popularity since unlike NMR and X-ray crystallography, it has the potential to visualize large, multi-subunit supramolecular complex without the requirement for a large volume of sample or crystallization. Luque and Castón have made an elaborated report on the use of Cryo-electron microscopy (Cryo-EM) to study dynamic processes of viral assembly using distinct conditions [131]. It could also reveal the information about substrate channeling within a multi-enzyme complex along with depicting the 3D structure of the complex [132, 133]. Computational modeling tools such as steered molecular dynamics simulation and molecular dynamic simulation along with binding energy calculations have been used to obtain mechanistic insights into the processivity of enzymes of the multi-enzyme complex [134].

Altogether, the above-described strategies, as well as examples of scaffold proteins, have gained popularity in the field of synthetic biology since such kinds of multi-enzyme systems not only improve overall fundamental understanding but also expand their applications as a cell-free system which would mimic the naturally occurring metabolic channeling processes.

## 6 Conclusion

The spatial organization of enzymatic cascades on suitable protein scaffold has rapidly gained attention as it resembles cellular multi-enzyme complexes where scaffolding plays an important role in substrate channeling to attain optimum catalytic efficiency and providing a stable microenvironment. Therefore, the natural concept of scaffolding enzyme cascades has been adapted with the integration of synthetic biology for the development of an artificial scaffold system. With regards to this, diverse proteins have been utilized for creating artificial scaffold systems based on their remarkable properties. Their results signify that promising progress in this field has been made. However, further investigation is requisite for designing scaffold systems for complex metabolic pathways as well as for optimization of the system for large-scale industrial applications. This will create more robust mega-enzyme assemblies which will broaden its applications in the field of biofuel and bioprocess technology, enzyme engineering, and biosensor.

## CRediT authorship contribution statement

**Shubhada Gad:** Formal analysis, Writing – original draft. **Sonal Ayakar:** Writing – review & editing, Writing – original draft, Supervision.

## Declaration of Competing Interest

The authors declare that they have no known competing financial interests or personal relationships that could have appeared to influence the work reported in this paper.
